# Nitrogen fixation and denitrification activity differ between coral- and algae-dominated Red Sea reefs

**DOI:** 10.1038/s41598-021-90204-8

**Published:** 2021-06-03

**Authors:** Yusuf C. El-Khaled, Florian Roth, Nils Rädecker, Arjen Tilstra, Denis B. Karcher, Benjamin Kürten, Burton H. Jones, Christian R. Voolstra, Christian Wild

**Affiliations:** 1 Marine Ecology Department, Faculty of Biology and Chemistry, University of Bremen, 28359 Bremen, Germany; 2 Red Sea Research Center, King Abdullah University of Science and Technology (KAUST), Thuwal, 23995 Saudi Arabia; 3 Baltic Sea Centre, Stockholm University, 10691 Stockholm, Sweden; 4 Faculty of Biological and Environmental Sciences, Tvärminne Zoological Station, University of Helsinki, 00014 Helsinki, Finland; 5 Department of Biology, University of Konstanz, 78457 Konstanz, Germany; 6 Laboratory for Biological Geochemistry, School of Architecture, Civil and Environmental Engineering, École Polytechnique Fédérale de Lausanne (EPFL), 1015 Lausanne, Switzerland; 7 Australian National Centre for the Public Awareness of Science, Australian National University, ACT, Canberra, 2601 Australia; 8 Project Management Jülich, Jülich Research Centre GmbH, 18069 Rostock, Germany

**Keywords:** Biogeochemistry, Community ecology, Ecophysiology, Ecosystem ecology, Biochemistry, Ecology, Microbiology, Physiology, Biogeochemistry, Climate sciences, Ecology, Environmental sciences, Ocean sciences

## Abstract

Coral reefs experience phase shifts from coral- to algae-dominated benthic communities, which could affect the interplay between processes introducing and removing bioavailable nitrogen. However, the magnitude of such processes, i.e., dinitrogen (N_2_) fixation and denitrification levels, and their responses to phase shifts remain unknown in coral reefs. We assessed both processes for the dominant species of six benthic categories (hard corals, soft corals, turf algae, coral rubble, biogenic rock, and reef sands) accounting for > 98% of the benthic cover of a central Red Sea coral reef. Rates were extrapolated to the relative benthic cover of the studied organisms in co-occurring coral- and algae-dominated areas of the same reef. In general, benthic categories with high N_2_ fixation exhibited low denitrification activity. Extrapolated to the respective reef area, turf algae and coral rubble accounted for > 90% of overall N_2_ fixation, whereas corals contributed to more than half of reef denitrification. Total N_2_ fixation was twice as high in algae- compared to coral-dominated areas, whereas denitrification levels were similar. We conclude that algae-dominated reefs promote new nitrogen input through enhanced N_2_ fixation and comparatively low denitrification. The subsequent increased nitrogen availability could support net productivity, resulting in a positive feedback loop that increases the competitive advantage of algae over corals in reefs that experienced a phase shift.

## Introduction

Nitrogen (N) is vital for all living organisms and is required for primary production and the production of biomass. Among the key elements required for life (i.e., N, carbon, phosphorus [P], oxygen and sulphur^1^), N in the form of dinitrogen (N_2_) gas has the greatest total abundance in the environment^2^. Ironically, however, N_2_ gas is the least accessible for flora and fauna^1^. In oligotrophic marine ecosystems such as coral reefs, primary production is limited by low amounts of bioavailable N forms such as ammonium (NH_4_^+^) or nitrate (NO_3_^-^)^3–5^. Yet, coral reefs belong to the most productive ecosystems on earth and are regarded as oases in an oceanic desert^6–8^. In this context, microbial N cycling plays a key role by introducing, recycling and removing N from coral reefs^9^. Particularly, biological N_2_ fixation, i.e., the conversion of atmospheric N_2_ into bioavailable NH_4_^+^ by prokaryotic microbes (diazotrophs), can alleviate N limitation for coral reef primary producers^10^. In addition, the recycling of de novo bioavailable N via nitrification^4,11^ may serve as a mechanism to prevent the loss of N^12^. In contrast, denitrification (i.e., the conversion of nitrate to atmospheric N_2_ by microbes) may remove bioavailable N in times of high environmental N availability^12–14^. Likewise, fixed N can be transformed into atmospheric N_2_ via anaerobic ammonium oxidation (ANAMMOX), a pathway functioning as an additional N removing mechanism in coral reef sponges^15^, and hypothetically in other coral reef associated organisms^12^. Whereas N influxes to coral reefs via N_2_ fixation are comparably well-studied^10,16,17^, knowledge about N efflux via denitrification is limited to some coral reef substrates (such as reef sediments)^13,14^, and is just starting to be generated for other coral reef organisms (e.g., hard corals)^18–21^. Corals reefs and their main ecosystem engineers, scleractinian corals, are adapted to nutrient-poor environments^22,23^. Under these conditions, both the import of bioavailable N via N_2_ fixation as well as the removal via denitrification may essentially contribute to maintaining a stable, low N availability and, hence, ecosystem functioning^22^. Further N cycling processes have been detected or hypothesised in coral reef environments^12^. For example, nitrification, i.e., the oxidation of NH_4_^+^ to nitrite (NO_2_^-^) and NO_3_^-^, has been measured in coral reef environments^4,24^, and may function as an internal recycling mechanism with nitrate serving as a substrate for coupled denitrification^12^.

Coral reefs not only belong to one of the most productive but also to the most threatened ecosystems on the planet. Global and local change associated stressors such as ocean warming and acidification^25,26^, eutrophication^27^, and overfishing^28^ undermine the health of coral reefs and can eventually lead to coral mass mortality^29^. The remaining coral skeletons offer substrates^30–32^ for fast-growing, highly competitive algae assemblages^33^, which may lead to a transition from coral-dominated to algae-dominated reef states. Due to the stability of these novel communities, these transitions have been interpreted as phase-shifts^34–36^. Although the effects of these phase-shifts on ecosystem services^37–41^ and functioning^42,43^ have received some attention, Williams and Graham^44^ emphasise our yet rudimentary understanding of alterations in coral reef functional ecology. Although we already observe different ecosystem states (e.g., coral-dominated or alternative states on coral reefs)^45^, our knowledge about their functioning is still in its infancy. Additionally, functioning likely differs between coral- and algae-dominated communities^46,47^.

N cycling is critical for the stability of coral reef ecosystems; however, it has not been investigated yet how gain (via N_2_ fixation) and loss (via denitrification) terms of bioavailable N differ quantitatively between coral- and algae-dominated reef states. For this study, we hypothesised differences between coral- and algae-dominated reef states in the amount of total fixed and denitrified N, based on differences in N_2_ fixation and denitrification activities of different benthic categories^10,48,49^. Hypothetically, an altered reef community structure associated with phase-shifts could lead to different total N_2_ fixation and denitrification budgets in coral- and algae-dominated reef areas. Further, we hypothesised that N cycling processes may have the potential to amplify and catalyse phase-shifts through the proliferation of turf algae as prominent N_2_ fixers^50^. The role of denitrification in reef communities is mostly unknown, as well as the interplay of both N_2_ fixation and denitrification in coral- and algae-dominated reefs. Understanding N cycling patterns in baseline scenarios (i.e., coral-dominated) and alternative reef states (i.e., algae-dominated) is, hence, of paramount interest to gain a holistic understanding of these dynamic systems, which then can be used as a basis to address, elaborate, and expand future management strategies.

In the present study, we carried out acetylene-based incubations (i) to identify the key players that import and/or remove nitrogenous compounds into/from the reef system; and (ii) to provide a relative budget for two counteracting N cycling processes (i.e., N_2_ fixation and denitrification) in a comparative framework that covers the main species of six key benthic categories that together account for > 98% of the benthic cover on a central Red Sea coral reef with two distinct reef areas.

## Results

### Benthic community composition

**Figure 1:**
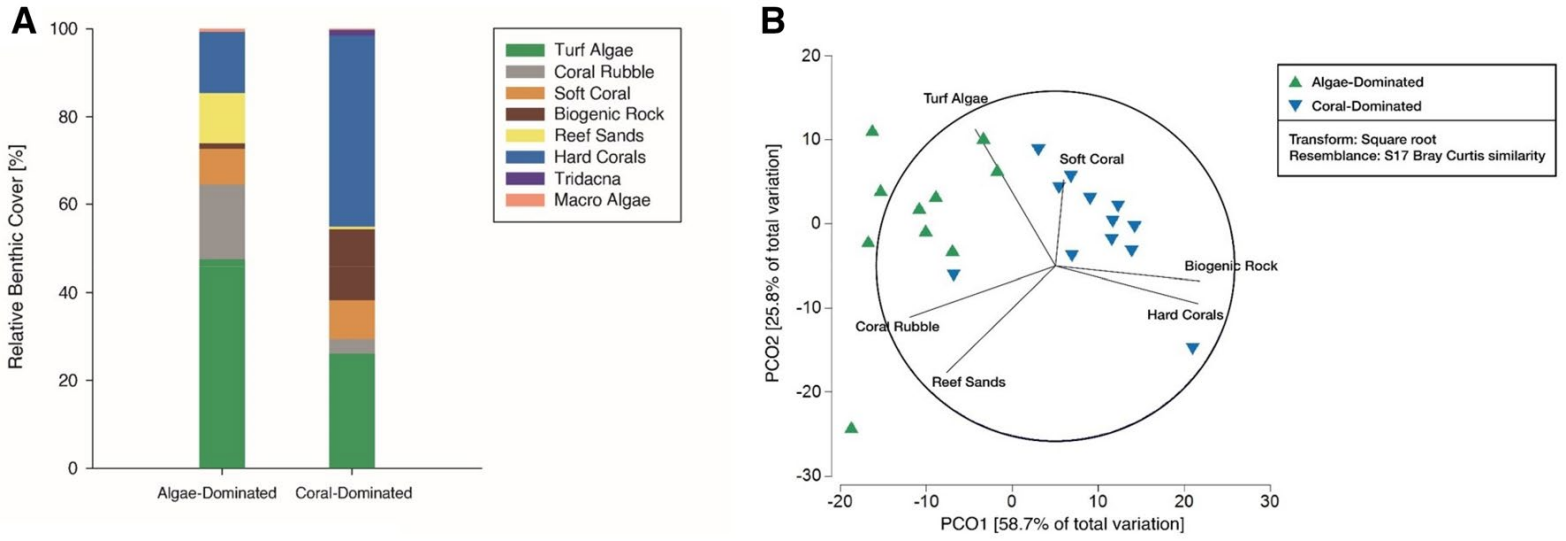
(**A**) Relative benthic cover in algae- and coral-dominated areas. Benthic cover is presented as mean proportional cover of major benthic categories assessed by photo quadrats (n = 10 in algae-dominated areas, n = 12 in coral-dominated areas). Adapted from Roth et. al ^51^. (**B**) Principle Coordinate Analysis (PCO) of benthic community cover at the algae-dominated (green) and coral-dominated (blue) areas. Vector overlays shows correlations > 0.4 based on Pearson ranking, green triangles display replicates of algae-dominated areas (n = 10), blue triangles display replicates of coral-dominated areas (n = 12).

Two distinct reef community states characterised by contrasting relative cover of benthic categories were identified (Fig. 1A, Fig. S1) and pre-defined as “algae-” and “coral-dominated reef areas” (according to Roth and others^51^). A detailed overview of the benthic communities can be found in Table S1. Briefly, algae-dominated areas displayed approximately twice as much turf algae-cover compared to coral-dominated areas (Mann–Whitney *U* *p* = 0.007), whereas hard coral cover was threefold lower (Mann–Whitney *U* *p* < 0.001). Pre-defined differentiation between algae- and coral-dominated reef areas were confirmed visually by Principle Coordinate Analysis (PCO, Fig. 1B) and ANOSIM (R = 0.605, *p* = 0.001). Hard corals and turf algae were the major drivers of separation and explained 64.2% of the dissimilarities between reef areas (DistLM).

### Nitrogen fluxes of individual benthic categories

**Figure 2:**
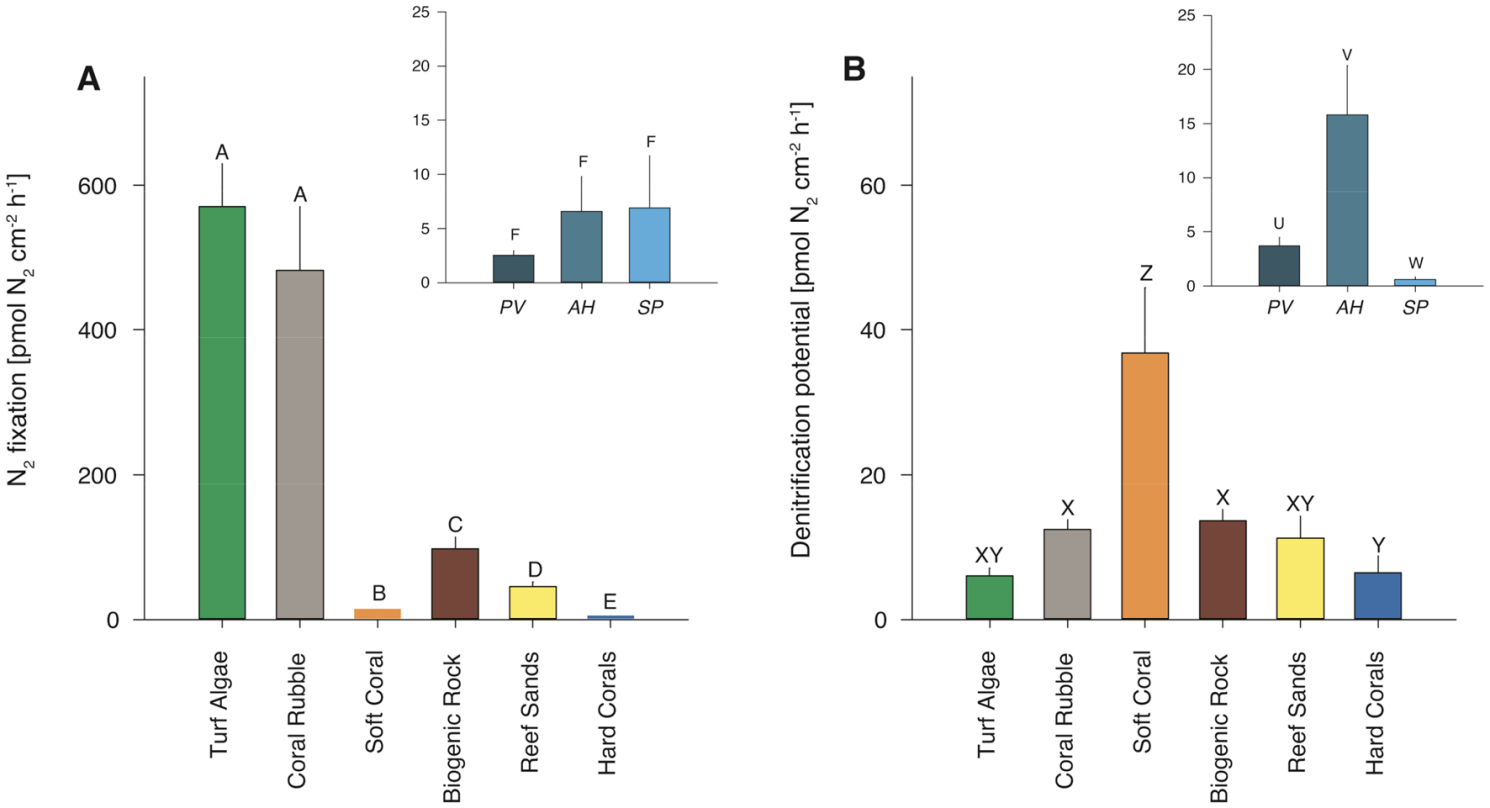
**(A) **Dinitrogen (N_2_) fixation rates and **(B)** denitrification potential of investigated benthic categories and hard coral species. Organisms and substrates (turf algae n = 5, coral rubble n = 4, soft coral n = 5, biogenic rock n = 5, reef sands n = 5, hard corals n = 13) were sampled randomly from both reef areas. Rates and potential for hard corals consist of mean values of *P. verrucosa* (PV, n = 5), *A. hemprichii* (AH, n = 4), *S. pistillata* (SP, n = 4). Letters above bars indicate significant differences if different, or non-significance if shared. Y-Axis labels for imbedded hard coral data plots are analogue to respective N_2_ fixation rate or denitrification potential plot. Note different scales for N_2_ fixation and denitrification.

Turf algae and coral rubble showed highest N_2_ fixation rates per substrate surface area, being approximately 5 to 6-fold higher than those of biogenic rock, 10 to 12-fold higher than reef sands, 32 to 39-fold higher than soft coral, and approximately two orders of magnitude higher than hard corals (Fig. 2A, Table 1 and S2).

**Table 1:**
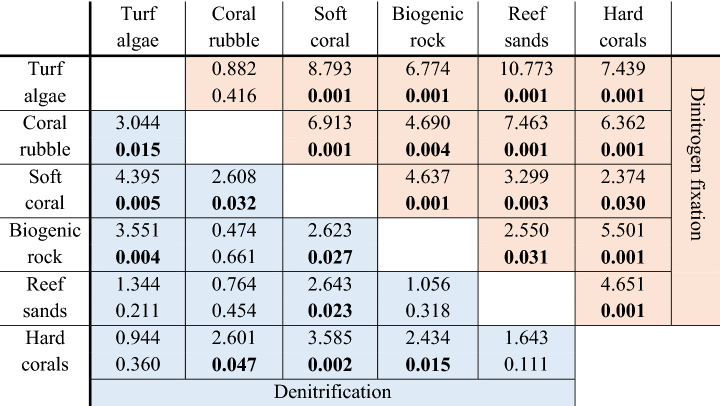
Results of permutational analysis of variance (PERMANOVA) and subsequent pair-wise tests for N_2_ fixation (highlighted in salmon) and denitrification (highlighted in blue) in all benthic categories.

Significant *p*-values in bold. Top: t-values; bottom: *p*-values.

Due to NO_3_^-^ addition during incubations, we present denitrification as potentials instead of rates (see Method section). The soft coral displayed the highest denitrification activity, being 3- to 4-fold higher than that of reef sands, biogenic rock and coral rubble, and 6-fold higher than denitrification potentials of hard corals and turf algae (Fig. 2B, Table 1 and S2). Among hard corals (i.e., data above comprised the average potentials across *P. verrucosa, A. hemprichii,* and *S. pistillata*), *A. hemprichii* showed the highest denitrification potential that was 5-fold higher than that of *P. verrucosa* (pair-wise PERMANOVA t = 3.407, *p* = 0.01) and 26-fold higher than *S. pistillata* (pair-wise PERMANOVA t = 5.696, *p* < 0.001).

### Nitrogen fluxes in different benthic categories referred to reef areas

**Figure 3:**
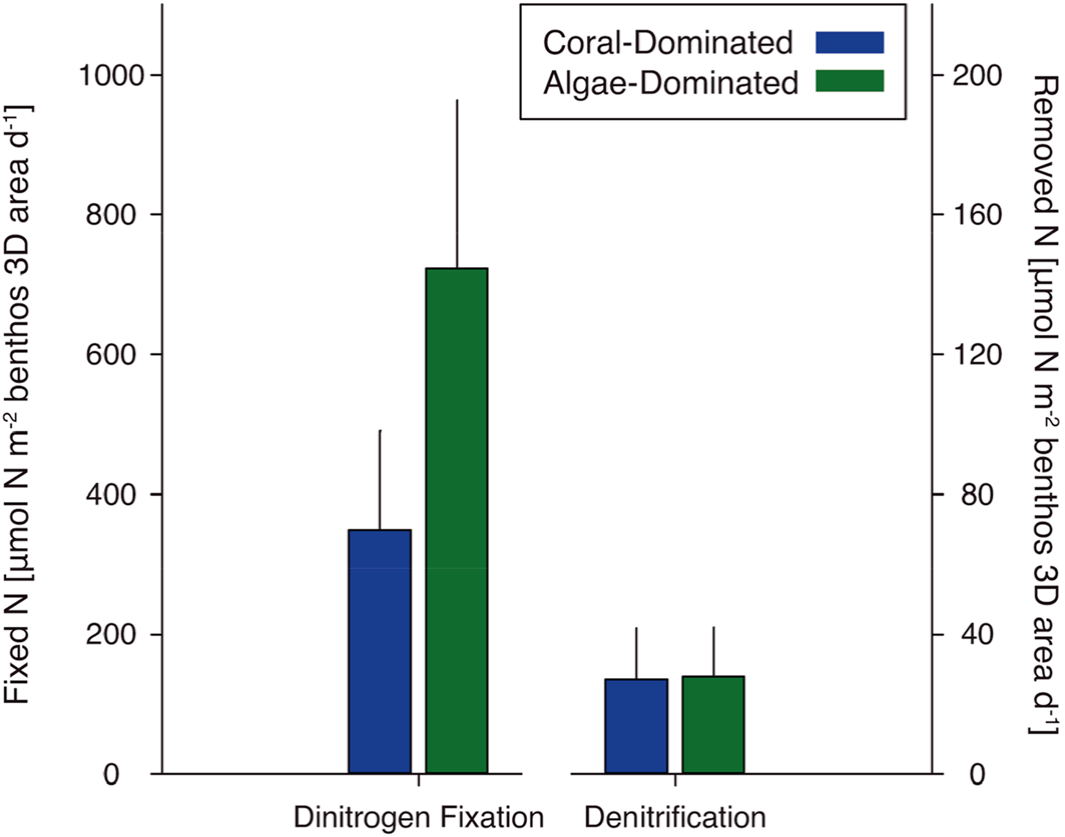
Total fixed (via dinitrogen fixation) and removed N (via denitrification) in both reef areas (calculated as sum of means of extrapolated rates of individual benthic categories ± standard propagated error; according to supplementary material SM3; no significant differences were observed by applying Friedman’s aligned rank test).

**Figure 4:**
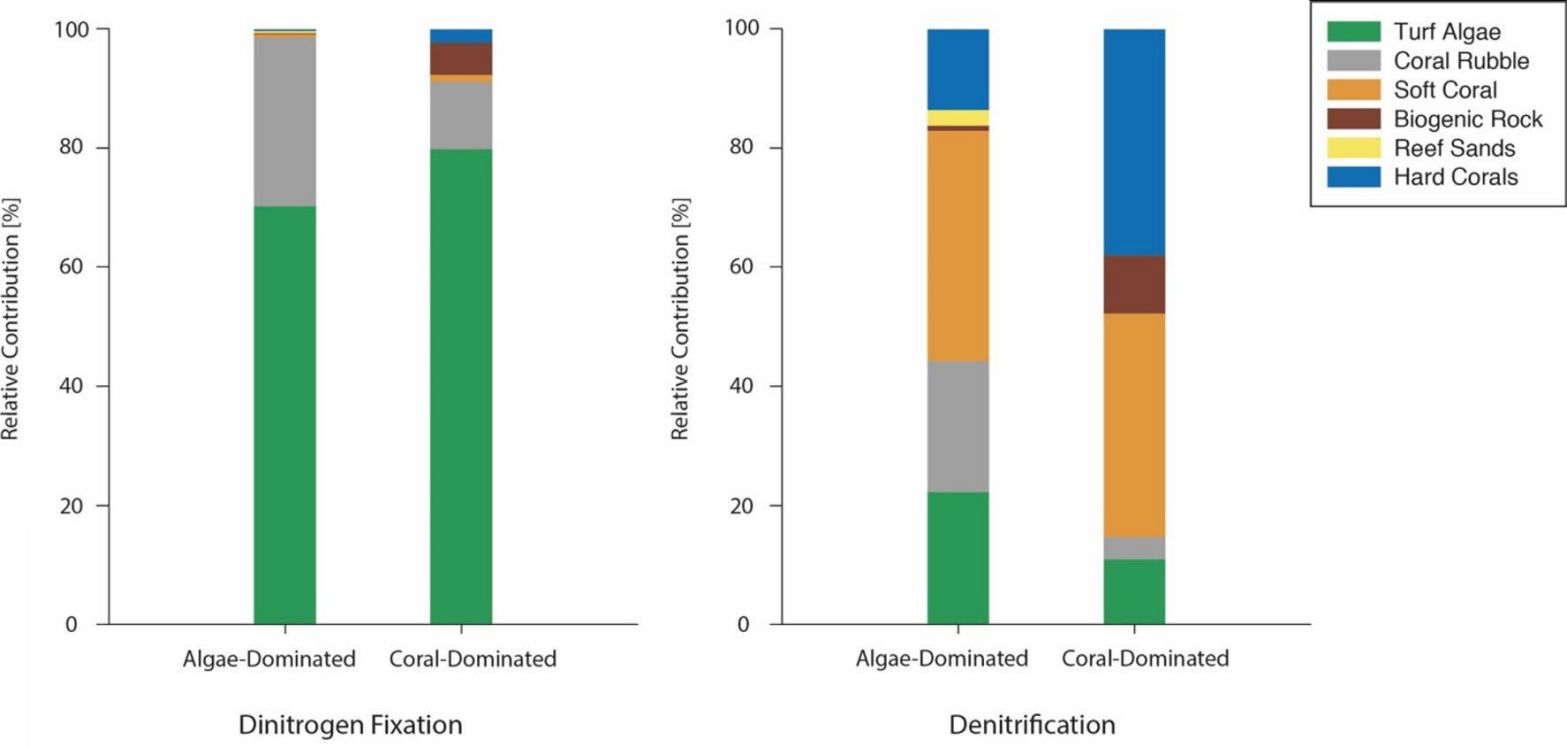
Relative contribution of studied benthic categories to total dinitrogen fixation and total denitrification in coral- and algae-dominated reef areas.

We used individual rates/potentials of N_2_ fixation and denitrification from all measured organisms and substrates (see Table S2) to calculate budgets for communities of the assessed reef areas dominated either by corals or turf algae. Cumulated fixed N was 2-fold higher in the algae-dominated compared to the coral-dominated area (Friedman’s aligned rank test *p* = 0.24), whereas denitrification was similar (Friedman’s aligned rank test *p* = 0.19; Fig. 3). Turf algae assemblages contributed most to N_2_ fixation in both algae-dominated (70.2%) and coral-dominated areas (79.8%), followed by coral rubble (28.4% in algae-dominated and 11.3% in coral-dominated area, respectively; Fig. 4). In contrast, hard and soft corals combined accounted for 75.5% and 52.4% of denitrification activity in coral- and algae-dominated areas, respectively (Fig. 4).

## Discussion

Stable and low N availability is of central importance to the health and resilience of coral holobionts^12,52^, and consequently, of coral reef ecosystems in general^22^. Thus, processes that introduce or remove bioavailable N, such as N_2_ fixation and denitrification, respectively, may play a key role in the functioning of coral reefs^22^. Our study is—to the best of our knowledge—the first to provide a comparative overview of denitrification activities of major coral reef related benthic categories. By investigating two N cycling process, we were able to extend previous work of Cardini and others^10^, who showed a link between reef-wide primary production and N_2_ fixation. We related benthic category-specific N_2_ fixation rates and denitrification potentials (per 2D substrate surface area, Table S2) to their relative contribution on a 3D level (Table S3), and likewise extrapolated metabolic processes to both coral- and algae-dominated reef communities (per 3D benthos surface area, Fig. 3, Table S1). This may be of particular importance, as algal dominance on coral reefs will likely increase as a consequence of frequently occurring mass coral bleaching events^53,54^ and reduced time available for recovery^55^ that diminish the return to coral-dominated reef states^56^.

### N_2_ fixation and denitrification by key benthic categories on coral reefs

Individual N_2_ fixation rates of benthic categories are in line with^10,20,48,49,57^ or lower^10,58,59^ than rates reported in previous studies (see Table 2) from the Red Sea that were obtained with similar measurement techniques (i.e., acetylene-based assays) and sampling intervals (t_0_ and t_24_). Slightly lower N_2_ fixation rates in the present study may a) be due to an underestimation caused by an initial lag phase associated with acetylene-based assays^60,61^ (see limitations), which was omitted from rate calculations of other studies^59^ but included here; or b) due to varying sampling times: benthic categories of the present study were incubated in March 2018, which is the winter season. It has been demonstrated that N_2_ fixation activity in winter is generally lower than in summer^10,49,62^, which could explain lower N_2_ fixation rates of the present study compared to previously reported rates (see Table 2). The effect of seasonality on the denitrification potential of our benthic categories remains unknown.

**Table 2:** N_2_ fixation rates (nmol N_2_ cm^−2^ h^−1^) of investigated benthic categories in comparison with values reported from other coral reef areas worldwide acquired via acetylene-based assays. All N_2_ fixation rates were converted with a conservative conversion factor of 4:1 (C_2_H_4_:N_2_) according to Mulholland et al.^157^. Values are presented in mean ± SE.

N_2_ fixation	Location	Reference
**Turf algae**		
0.57 ± 0.05	Central Red Sea	Present study
0.44 ± 0.04*	Northern Red Sea	Rix and others^49^
2.31 ± 0.09**	Northern Red Sea	Shashar and others^59^
**Coral rubble**		
0.48 ± 0.09	Central Red Sea	Present study
1.00 ± 0.25	Great Barrier Reef, Australia	Davey and others^75^
0.58 ± 0.20*	Northern Red Sea	Cardini and other^10^
0.90–4.00	Great Barrier Reef, Australia	Larkum^76^
0.74–5.70	Great Barrier Reef, Australia	Larkum and other^17^
13.86 ± 4.11**	Northern Red Sea	Shashar and others^59^
**Soft Coral (*Xenia* sp.)**		
0.014 ± 0.004	Central Red Sea	Present study
0.003 ± 0.000*	Northern Red Sea	Bednarz and others^48^
**Biogenic Rock**		
0.098 ± 0.016	Central Red Sea	Present study
0.112 ± 0.038*	Northern Red Sea	Rix and others^49^
0.13 ± 0.01**	Northern Red Sea	Shashar and others^59^
**Reef Sands**		
0.046 ± 0.006	Central Red Sea	Present study
0.296 ± 0.067*	Northern Red Sea	Bednarz and others^58^
4.88 ± 1.38**	Northern Red Sea	Shashar and others^59^
**Hard Corals**		
0.005 ± 0.002	Central Red Sea	Present study
0.000–0.003	Central Red Sea	Pogoreutz and others^57^
0.012 ± 0.003*	Northern Red Sea	Cardini and others^10^
0.016 ± 0.087	Central Red Sea	Tilstra and others^20^
0.988 ± 0.211**	Northern Red Sea	Shashar and others^59^

*Winter season; **Summer season.

Our results suggest a clear distinction between key benthic categories, in which the most active N_2_-fixers showed lowest denitrification potentials and vice versa. Turf algae and coral rubble were identified as the largest N_2_-fixers, confirming previous findings by Cardini and others^10^; however, they play a minor role in reducing N availability via denitrification. Reef sands, as active sites of microbial N transformations in coral reefs^14^, played only a marginal role in both N_2_ fixation and denitrification in the present study, compared to the other benthic categories. Our comparatively low N_2_ fixation and denitrification rates in reef sands may have been a result of well-oxygenated top sediment layers^63^, which could inhibit these anaerobic processes^64,65^. Even though reef sand N cycling rates were likely underestimated in the studied reef areas (as top layers were sampled; see limitations section), reef sands solely cover a minor fraction of the benthic cover.

From an ecological perspective, turf algae are pioneers^33^ and opportunists^66,67^, which can form extensive mats even under oligotrophic conditions^68,69^. Furthermore, they can rapidly take over bare substrate due to high growth rates^33,66,70,71^. The high N demand needed to fuel metabolism and biomass production in filamentous turf algae can, to a large extent, be satisfied by high N_2_-fixing activities^33,49,72,73^. A recent study showed that accumulated N in the form of turf algal biomass can be exported to the wider reef^33^. N_2_ fixation activities of coral rubble were similar to those of turf algae, which confirms findings of a recent study^74^. Coral rubble N_2_ fixation activity measured here is in the range of previous studies^75,76^ and may, thus, be driven by microbial communities inhabiting coral rubble surfaces, as suggested by Davey and others^75^, who observed significantly higher N_2_ fixation activity in coral rubble compared to living hard corals.

Among all benthic categories, corals (both hard and soft corals) showed lowest N_2_ fixation activities, being in a similar range^10,48,77^ or significantly lower^59^ than rates measured previously with acetylene reduction techniques. Lower N_2_ fixation rates for hard corals of the present study may have occurred due to a phosphate limitation of N_2_ fixation^13,78,79^, as the estimated N:P ratio of 83:1 in the incubation water (see Methods) ranged above the canonical Redfield ratio of 16:1^80^. Additionally, discrepancies could also result from differing surface area determination techniques used here and in other studies. Previous studies determined the surface area of investigated benthic categories, such as hard corals, using the aluminium foil method^81^ or advanced geometry^49,58^, but potentially lack accuracy^82^ and, hence, potentially underestimate the surface area of hard corals and, thus, overestimate N fluxes. The soft coral displayed highest denitrification activity. The interplay of N_2_ fixation and denitrification, favouring low internal N availability, may be vital for the stability between the coral host and the associated photosynthetic algal endosymbiont of the family Symbiodiniaceae (sensu Rädecker and others^12^). Low N_2_ fixation and comparatively high denitrification activity may promote the host’s control over symbiotic algae^83^ by keeping N availability limited^5,84^. As such, seasonal fluctuations ^85^ or environmental stressors, like eutrophication or ocean warming, may influence both processes^12^, leading to shifts from N to P or another micronutrient (e.g., iron) limitation^86–88^, which can result in a breakdown of the coral-Symbiodiniaceae symbiosis^87^. Furthermore, Tilstra and others^20^ hypothesised a negative correlation between heterotrophic capacity and denitrification activity in coral holobionts, as previously suggested for diazotrophs^77^. The hard coral species used in the present study are all on the autotrophic end of the mixotrophic spectrum^89–92^. As such, these coral species rely mostly on the Symbiodiniaceae for their energy. As denitrifying microbes are heterotrophic^93,94^, it is likely that photosynthates translocated from the Symbiodiniaceae are their main source of energy. Thus, a differing heterotrophic capacity of the coral could potentially influence denitrification potentials. This hypothesised link may also explain the high denitrification activity found in the investigated soft coral of the family Xeniidae, which is a functional autotroph^95^.

Finally, denitrification is an anaerobic process. Potentially, observed denitrification potential differences among the hard coral species and *Xenia* sp. occurred due to their different capacity in releasing and producing mucus^96,97^, with thick mucus layers favouring high anaerobic potentials that might facilitate denitrification^96^. It has been demonstrated recently that soft coral mucus does not provide a favourable habitat for associated diazotrophs^97^, which in return could explain observed comparatively low N_2_ fixation rates.

### Implications for coral- and algae-dominated reefs

Extrapolated reef-wide fixed N via N_2_ fixation of both coral- and algae-dominated reef areas (350.01 ± 97.87 and 722.91 ± 241.26 µmol N m^−2^ d^−1^, respectively; Fig. 3) is in line with previously calculated reef-wide N_2_ fixation budgets of Larkum and others^17^ and Cardini and others^10^, who have reported an annual average N_2_ fixation activity of 156–1330 µmol N m^−2^ d^−1^ and 546 ± 69 µmol N m^−2^ d^−1^, respectively.

At the community level, the relative contribution of key benthic categories varied when compared to N fluxes related to substrate surface area. Together, turf algae and coral rubble represent 91% of overall N_2_ fixation in coral-dominated and 99% in algae-dominated reef areas. Hard and soft corals are key players in coral-dominated reef areas, in which both benthic categories together account for 52% of benthic cover (in the investigated Abo Shosha reef area) and contributed equally to the 78% of overall denitrification. This is surprising, as both benthic categories showed lowest (hard corals) or highest (soft coral) denitrification activity. While all investigated hard coral species showed similar N_2_ fixation patterns, findings regarding their denitrifying activity showed a large variability (Fig. 2). Based on our results, we hypothesise that reefs with a higher *Acropora hemprichii* cover (compared to *Stylophora pistillata* or *Pocillopora verrucosa*) display higher capacities to remove bioavailable N via denitrification and could, thus, be more resilient to higher N availability than those of *S. pistillata* or *P. verrucosa* dominance. However, we have considered only a small selection of hard corals with a branching morphology that are considered as autotrophs^89–92^. It remains speculative how other hard coral species, such as those of mounding and plating morphologies, or heterotrophic corals^77,98^ with different mass transfer characteristics^99,100^ contribute to potential reef resilience.

Besides being most abundant in coral-dominated areas, hard corals (especially branching hard corals, represented by the species selected in the present study) contribute most to the three-dimensional structure (i.e., spatial complexity or rugosity) of coral reef ecosystems^100–103^, which increases the relative importance of hard corals for N_2_ fixation and particularly denitrification in both reef areas in the present study. A reduction of hard coral cover and, thus, spatial complexity (i.e., a loss of cryptic 3D area) also leads to a reduced contribution to overall denitrification (Fig. 4), even though denitrification potentials for hard corals are similar to those of reef sands and turf algae when related to 2D substrate surface area (Fig. 2). Even in the algae-dominated reef area, both hard and soft corals still contributed to more than 50% of overall denitrification (Fig. 4), despite covering only ~ 22% of the seafloor. The mentioned reduction or loss of spatial complexity is commonly associated with coral-phase shifts^104^. Whereas the importance of structural complexity and consequences of its loss in coral reefs has been recognised on multi-fold levels before, e.g., on fish communities^103–107^, invertebrate diversity^108,109^ and ecosystem services^41,102,110,111^, results of the present study also suggest substantial consequences on N cycling dynamics. The most striking result was the difference in total N import via N_2_ fixation in both reef states, as well as changes in the relative contribution of coral-associated denitrification in the respective reef areas. Turf algae were identified as key N_2_-fixers and their higher abundance in benthic coverage leads to a 100% increase of N_2_ fixation in algae-dominated compared to coral-dominated areas (Fig. 3). At the same time, denitrification, a process that may alleviate coral reef environments from excess N^12–15,20^, remained stable in the algae-dominated part of the reef. Future studies should determine the role of ANAMMOX in coral reefs, as ANAMMOX may play a vital role in removing bioavailable N from coral reef environments^12^. However, key ANANMMOX-performing players have not been identified in coral reefs yet, nor were they included in the present study (see limitations). Nevertheless, a higher N availability (i.e., higher fixed N inputs with stable denitrification activity) in algae-dominated reefs could have multiple consequences resulting in a positive feedback loop. Just as eutrophication promotes turf algae growth on coral reefs^112^, higher N accumulation from N_2_ fixation could relieve N limitation and cause algae to proliferate in nutrient-poor waters^113,114^. This can result in high abundances of benthic algae that in turn deter herbivorous fish that successively control algal proliferation by grazing^115^. Jessen and Wild^116^ have described this feedback loop before, and we here append that higher N availability (via N_2_ fixation) in algae-dominated reefs can be further utilised for algal growth or metabolism. Subsequently, increased N availability could facilitate the release of algal exudates such as dissolved organic nitrogen (DON)^117^ from benthic algae during active growth or decomposition. Ultimately, this feedback loop could turn a reef from a previously net sink of DON into a DON source, which similarly occurs during eutrophication events^16,118^.

### Implications for alternative reef states

In the present study, we evaluated two distinct reef communities and the implications of their varying benthic composition. Microbial communities associated with the benthic categories likely not only vary between coral- and algae-dominated reefs^119^, but also between investigated benthic categories^119–122^. Although the microbial community composition was not considered in this study (see limitations), varying microbial communities and their interactions might have implications for N_2_ fixation and denitrification activities and should be investigated in future studies. Discontinuous shifts^123^, shifts of varying intensity^124^, or community shifts from reefs dominated by corals to assemblages other than benthic (turf) algae have been reported (reviewed in Norström and others^45^). Aside from algae-dominated reefs, alternative reef states can be dominated by corallimorphia, sponges, ascidians, or soft corals^45^. Particularly, soft coral dominance is common in the Red Sea^125,126^ and other regions such as Taiwan, East Pacific^127^ and at the Great Barrier Reef, Australia^128^, where the soft coral genus *Xenia* attains as much as 80% of benthic cover after disturbance^45,129^. Moreover, climate change associated stressors such as ocean acidification might induce community shifts from hard to soft coral dominance^130^. Bednarz and others^48^ propose that soft corals may become an important player in N cycling due to their increasing dominance in benthic cover in the northern Red Sea. Based on the results of the present study, we carefully speculate that a high soft coral cover may alleviate degraded reefs, or reefs in a transitional state, from excessive N. The extent to which *Xenia* sp., as the main denitrifying organism, can decelerate coral reef degradation remains to be determined in future studies. Hypothetically, N limitation could be exacerbated in *Xenia* sp. dominated reef areas, as more N is removed via denitrification than in hard coral or algae-reef dominated areas. This hypothesis is supported by findings of Pupier and others^97^, who suggest a significant decrease in N_2_ fixation and subsequent N limitation in soft coral-dominated reefs.

Coral rubble-dominated areas, particularly reef flats, can be the result of fragmentation and erosion processes of nearby reef areas such as leeward slopes, resulting in mobile fragments that are unsuitable for coral colonisation^131^. Findings of the present study confirm previous hypotheses, in which the potential of coral rubble as highly active N_2_-fixers can aggravate N influxes to a destroyed or vulnerable reef^52,75^. At the same time, our findings indicate that the denitrifying capacity of rubble-dominated reefs is presumably lower than in intact coral-dominated areas (i.e., due to lower structural complexity and relatively lower denitrification activity compared to soft corals), which suggests that this reef state could compound N influxes, resulting in N accumulation at the ecosystem scale.

We demonstrated that N influxes via N_2_ fixation could lead to an aggregation of N in algae-dominated communities, while differences regarding the bioavailable N removal via denitrification were not observed between coral- and algae-dominated reef areas. Potentially, N_2_ fixation rates^10,49,58^ as well as denitrification potentials (^85^) experience seasonal fluctuations that might cause feedback responses in the extrapolated fixed or removed N. The range of these feedback responses remains to be determined.

N cycling processes in coral reefs, including anaerobic ammonium oxidation (ANAMMOX, transformation of fixed NH_4_^+^ and NO_2_^−^ to elemental N_2_), that potentially remove fixed N from the system^132,133^ have been identified in coral microbiomes^134^ and coral-reef sponges^15^. The extent to which ANAMMOX can serve as a N removal process in shifting reefs remains to be determined. Further, cryptic habitats in coral reefs harbouring high abundances of sponges^135^ may reduce bioavailable N via ANAMMOX and should be prioritised in future studies, as they were not considered in the present study (see limitations). The biogeochemical significance of stressor-induced phase shifts and the resulting loss of structural complexity (i.e., cryptic habitats) in reef N cycling remain unknown and should be the subject of future studies.

## Material and methods

### Study site and benthic community composition

The Abu Shosha reef in the Jeddah Region (22° 18′ 15″ N, 39° 02′ 56″ E) on the west coast of Saudi Arabia in the central Red Sea was chosen due to the co-occurrence of both coral- (i.e., > 40% hard coral cover) and algae- (< 15% hard coral cover, > 40% turf algae cover) dominated areas within the same reef (Fig. S1)^51^. Both areas are approx. 50 m^2^ in size, were located at the same water depth (~ 5 m) and were solely used as a base for relating the respective N cycling rates to the reef area. Specimens were sampled from the total reef area (see next paragraph). The selected reef displays a small-scale heterogeneity of communities and varying degrees of community composition with both target reef areas being less than 30 m apart from each other.

Benthic community composition of the Abu Shosha reef was determined for an earlier study^51^ by photo quadrats^136^, providing a two-dimensional (2D) planar reef coverage of each benthic category. Briefly, a PVC quadrat (50 × 50 cm, 0.25 m^2^) was randomly placed on the reef surface (12 × in coral-dominated area, 10 × in the algae-dominated area), and a photograph was taken from approx. 1 m distance to the substrate. Photographs were then analysed with the software Coral Point Count with Excel extension (CPCe) 4.1^137^. With the help of the software, 48 randomly located points were overlaid on the photographs, resulting in 576 and 480, respectively, data points per study area. The underlying benthos for each data point was determined to the lowest possible taxon. The major benthic categories of the investigated reef were (Fig. 1A): filamentous turf algal assemblages (hereafter termed turf algae), coral rubble, soft coral (i.e., *Xenia* sp.), biogenic coral rock (hereafter termed biogenic rock), carbonate reef sands (hereafter termed reef sands), hard corals, macroalgae, and the giant clams (*Tridacna* sp.). Hard corals were identified to the genus level. Turf communities consisted of a heterogeneous assemblage of different filamentous algae and cyanobacteria. Examples from the Northern Red Sea have shown that turf algae, as defined for the present study, account for the highest fraction (up to 90%) of benthic algal cover^138^. Areas of bare coral rock, that were not covered with any of the other benthic categories but associated with endolithic algae and crustose coralline algae were defined as biogenic coral rock^139^ (hereafter termed biogenic rock). Coral rubble was defined as dislodged parts of framework builders with its associated microbial community according to Rasser and Riegl^131^.

### Sample collection and maintenance

**Figure 5:**
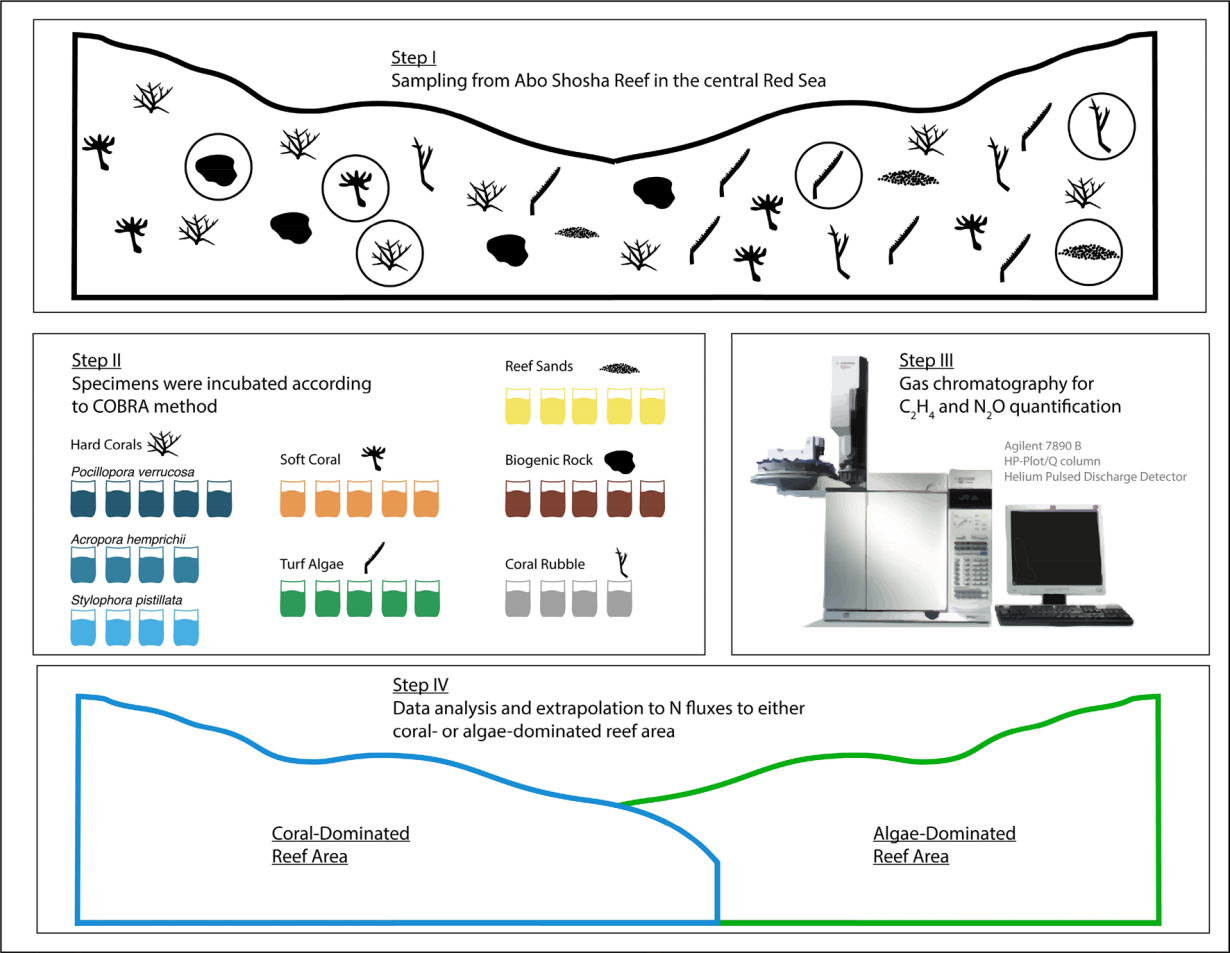
Stepwise illustration of sampling, data acquisition and analysis. Step I illustrates sampling of specimens randomly from the overall reef area (i.e., regardless of coral- or algae-dominated area). Afterwards (step II), all specimens were incubated according to the COBRA method^74^ with the displayed replication. Gas samples were taken for ethylene (C_2_H_4_) and nitrous oxide (N_2_O) measurements via gas chromatography (step III) for N cycling rate quantifications. Obtained data were used for extrapolation to N fluxes to either coral- or algae-dominated reef areas (step IV).

All benthic categories, i.e. hard corals *Pocillopora verrucosa* (n = 5), *Acropora hemprichii* (n = 4), *Stylophora pistillata* (n = 4), soft coral of the *Xenia* genus (n = 5), biogenic rock (n = 5), coral rubble (n = 4), reef sands (n = 5) and turf algae (n = 5), were collected randomly from the overall reef area (i.e., regardless whether from coral- or algae-dominated areas; Fig. 5—Step 1) and immediately incubated after sampling in March 2018. Due to feasibility, the three most abundant hard coral species were chosen as they represent the most abundant species of the Abo Shosha reef. To increase readability and comprehensiveness, we refer to “hard corals” from here on. The aforementioned benthic categories were selected as they comprised more than 98% of the benthic cover in both coral- and algae-dominated parts of the reef^51^. Where necessary, fragments were collected with hammer and chisel. *P. verrucosa*, *A. hemprichii*, *S. pistillata* and turf algae fragments (limestone covered with turf-algae) were approx. 10 cm long. Hard coral fragments were sampled from different coral colonies (> 10 m distance between each other) to account for genetic diversity. Individual coral colonies of *Xenia* sp. were collected with a small piece of anchoring rock (< 0.5 cm diameter) to prevent tissue damage. Reef sands were sampled using a Petri dish (polystyrene, 5.5 cm diameter, 1.4 cm depth) which was pushed carefully into the sand. Reef sands were then fixed to the dish from underneath so that upper sand “cores” with a max. sediment depth of 14 mm were sampled, covering a similar depth as reported previously^10,58^. All fragments and Petri dishes containing reef sand samples were immediately transferred to recirculation aquaria on the boat after sampling, each filled with ambient seawater. Fragments of *P. verrucosa*, *Xenia* sp., turf algae, coral rubble, biogenic rock as well as sediment samples were kept at ambient water temperature and light conditions until the experimental incubations started within 3 h after sampling. For *P. verrucosa*, it has been demonstrated successfully that freshly collected fragments can be utilised for physiological quantifications^140^. Fragments of *A. hemprichii* and *S. pistillata* were sampled two weeks prior all other specimens. After being transferred to the wet lab facilities of the Coastal and Marine Resources (CMOR) Core Lab at KAUST, fragments were distributed randomly into four independent replicate 150 L flow-through tanks (flow-through rate 300 L h^−1^) for two weeks to allow for acclimation and healing of tissue damage. Each aquarium was constantly supplied with ambient, sediment-filtered reef water from inshore reefs located 1.5 km off KAUST, and ambient light conditions, i.e., a photon flux of ~ 200 µM quanta m^−2^ s^−1^, representing the daytime average photon flux of the studied reef and water depth during this period of the year.

### Nitrogen cycle fluxes

Incubations were performed using a COmbined Blockage/Reduction Acetylene assay (hereafter COBRA; Fig. 5—Step 2) modified after El-Khaled and others^74^. Briefly, COBRA incubations were performed in gastight 1 L glass chambers (800 mL seawater + 200 mL headspace). As acetylene inhibits the production of NO_3_^-^ via nitrification^141,142^, seawater (ambient NO_3_^-^ concentrations ranged between 0.09 and 0.34 µM)^21,112^ was supplemented with nitrate to a final concentration of 5 µM as a substrate for the denitrification pathway to counteract substrate limitation (see supplementary material SM 5). Incubations with nitrate amended seawater have been performed successfully in previous studies^141–145^. Potentially, the addition of nitrate suppresses N_2_ fixation^146^, particularly as nitrate uptake has been reported for various benthic categories^147,148^. Theoretically, this could alter microbial functioning of benthic categories, as the acquisition of N via uptake is less cost-intensive^149^. Additionally, this results in an estimated NO_3_^-^:P ratio ranging between 5.09:0.06 and 5.34:0.07 in the incubation water, which is well above the canonical Redfield ratio of 16:1 (DIN:DIP)^80^. However, persistent N_2_ fixation rates in the presence of nitrate of up to 30 µM have been reported^149^. Furthermore, El-Khaled and others^74^ conclude that the technique provides sufficient information about the relative importance of different benthic categories by accounting for relative changes in N cycling rates (both N_2_ fixation and denitrification). COBRA provides denitrification “potentials” as artificially provided nitrate during the incubations drives denitrification above natural levels^74^. Acetylene was added to both incubation water and headspace at a concentration of 10%. This saturated acetylene concentration in the gastight incubation chambers leads to the preferential reduction of acetylene to ethylene (C_2_H_4_) instead of N_2_ to NH_4_^+^ by the key enzyme nitrogenase^150,151^. Moreover, acetylene blocks the nitrous oxide (N_2_O) reductase activity in the denitrification pathway leading to an accumulation of N_2_O^152,153^. Replicate samples were incubated and two additional chambers without specimens served as controls to correct for planktonic background activity. All incubations lasted for 24 h with a 12:12 h dark/light cycle and a photon flux of ~ 200 µM quanta m^−2^ s^−1^, representing the daytime average photon flux of the studied reef and water depth during this period of the year. Incubation chambers were submerged in a temperature-controlled water bath at 27 °C (resembling the ambient seawater temperature measured at the reef in 5 m depth during sampling) and constantly stirred (500 rpm) to ensure sufficient exchange between the water body and headspace. Gas samples were taken at the start (t_0_) and the end (t_24_) of each incubation, and analysed targeting C_2_H_4_ (as a proxy for N_2_ fixation) and N_2_O (as a proxy for denitrification) by gas chromatography and helium pulsed discharge detector (Agilent 7890B GC system with HP-Plot/Q column, lower detection limits for both target gases were 0.3 ppm). A detailed description of N_2_ fixation and denitrification rate/potential calculation can be found in the supplementary (SM 6). Briefly, results were normalised to incubation time, corrected for the seawater control signal, related to incubation volume, and normalised to the surface area of the organisms/substrates. Surface areas of incubated organisms/substrates were determined photometrically using cloud-based 3D models (Autodesk Remake v19.1.1.2)^154,155^ of *P. verrucosa*, *A. hemprichii*, *S. pistillata*, *Xenia* sp., biogenic rock, coral rubble and turf algae fragments. Reef sand surface areas were calculated using dimensions of Petri dishes that were utilised for sand core sampling (surface area = π * radius^2^). Notably, sediment depth and, hence, oxygenation status as well as pore-water movement through the sediment matrix affect the biogeochemical cycling, with potentially higher N cycling activity due to anaerobic milieus provided in deeper sand layers^63^. Oxygen fluxes were quantified parallel with identical benthic categories to validate that neither hypoxia nor hyperoxia (conditions that, e.g., are detrimental to organisms evoking alteration of physiological responses) conditions occurred during N cycling incubations^156^. We refer to supplementary material SM 7 for further information.

### Data treatment of nitrogen fluxes

Production rates of C_2_H_4_ and N_2_O were converted to N fluxes using conservative molar ratios of N_2_O:N_2_ = 1 and C_2_H_4_:N_2_ = 4^157^. Extrapolations for total fixed or removed N (via N_2_ fixation and denitrification, respectively) were performed according to formulas in supplementary material SM 8. Briefly, rates were extrapolated by multiplication according to reef benthos 3D area considering the respective 2D to 3D conversion factor (Table S4, according to Cardini and others^10^). Then, these benthic category-specific rates were used to account for the relative cover (i.e., 2D planar coverage obtained from cover assessments described previously) of each benthic category in the respective reef area (i.e., coral- and algae-dominated, resp.), which provides cumulative N fluxes related to 3D reef area (Fig. 4; expressed as fixed or denitrified µmol N m^−2^ benthos 3D area d^−1^, resp.).

### Statistical analysis

Statistical analyses were performed using Primer-E v6^158^ with the PERMANOVA + extension^159^. Differences in the N cycling processes among benthic categories were tested for significance using permutational analysis of variance (PERMANOVA) on a Bray–Curtis similarity matrix of square-root transformed data. In case significant differences occurred, pairwise t-tests with parallel Monte Carlo tests were performed. Type III (partial) sum of squares was used with an unrestricted permutation of raw data (999 permutations). Unless mentioned otherwise, hard coral data consists of pooled replicates of the three investigated hard coral species (i.e., *P. verrucosa*, *A. hemprichii*, *S. pistillata*) of which mean rates and standard error of means were calculated.

Normality (Shapiro–Wilk test) and differences between benthic category coverage in both reef areas (e.g., hard coral cover in coral-dominated versus hard coral cover in algae-dominated areas) were tested using SigmaPlot (Version 12.0). Two-tailed t-tests were used if data were distributed normally whereas Mann–Whitney-Rank-sum tests were used if data were not normally distributed. Differences in the benthic composition among reef areas (i.e., coral vs. algae-dominated) were visualised using a principal coordinate analysis (PCO). Total fixed and removed N in both reef areas were calculated based on the sum of means of extrapolated rates/potentials of individual benthic categories and the respective standard propagated error, with Friedman’s aligned rank test checking for significant differences among respective reef areas (using R v4.0.4^160^ with the interface Rstudio v1.0.153^161^). A one-way analysis of similarities (ANOSIM; 999 permutations) was used to describe the dissimilarities between both reef areas. Furthermore, a distance-based linear model (DistLM; 999 permutations) using a step-wise selection procedure with AICc as a selection criterion was used to calculate which benthic category(ies) coverage explained visualised dissimilarities best^158,159^.

### Limitations

Typical budget uncertainties include i) deviations from theoretical molar ratios (i.e., N_2_O:N_2_ and C_2_H_4_:N_2_) in different benthic categories (Wilson and others (2012) and references therein^162^), ii) methodological underestimation of N cycle processes^74,163,164^, iii) environmental alterations by benthic categories as an effect of benthic primary productivity^119^, iv) underestimations of N cycling rates for benthic categories that could not be assessed in their entirety such as reef sands, and v) reef-wide underestimations/omission of metabolic processes in cryptic habitats, such as cracks and crevices within the natural reef matrix, that harbour specific organisms (e.g., sponges, bryozoan, and tunicates). These organisms are generally not included in ex situ budget derivations. Nevertheless, the results of the present study are comparable to reefs of similar character and structural complexity. However, N cycling activity may vary substantially in reefs of differing structure, with more/fewer cracks and crevices in the reef matrix with inhabiting species that can remarkably contribute to metabolic processes in coral reefs^165,166^.

Furthermore, all specimens were sampled randomly from the overall reef area, thus, disregarding potential differences in the microbial communities of benthic categories between reef areas. These differences are likely to occur between coral- or algae-dominated reef areas^119^, and may influence N cycling processes. Potential effects of these differences, however, are rather insignificant as they would not allow for identification of differences in N cycling activities between benthic categories. Hence, we assume that shifts in the community composition are likely more relevant for the overall N fluxes than changes of N cycling activities within single benthic categories.

We, thus, consider the major results of the present study as solid and reliable, especially as the investigated benthic categories of investigated species cover > 98% in both reef areas. Nevertheless, we consider discussed and presented data as conservative estimates. Furthermore, aforementioned underestimation of N fluxes result from initial lag phases in the evolution of C_2_H_4_^60,74^, and from potential incomplete blockages of the denitrification pathway^163^. Due to potential underestimations of both N_2_ fixation and denitrification, we, thus, refrained from directly comparing both pathways.

## Supplementary information

Supplementary Information.
