# Supplementary material for: Nitrogen fixation and denitrification activity differ between coral- and algae-dominated Red Sea reefs
Source: Sci Rep. 2021 Jun 3;11:11820. doi: 10.1038/s41598-021-90204-8 (PMC8175748; doi:10.1038/s41598-021-90204-8)
Supplement: Supplementary file 1 — Supplementary Information. [file 41598_2021_90204_MOESM1_ESM.docx]

Supplementary Material (SM) to:

**“Nitrogen fixation and denitrification activity differ between coral- and algae-dominated Red Sea reefs”**

Yusuf C. El-Khaled^1*^, Florian Roth^2,3,4^, Nils Rädecker^2,5,6^, Arjen Tilstra^1^, Denis B. Karcher^1,7^, Benjamin Kürten^8^, Burton Jones^2^, Christian R. Voolstra^2,5^, Christian Wild^1^

*corresponding author: yek2012@uni-bremen.de

**SM 1 – Photos showing distinct coral- and algae-dominated reef areas.**


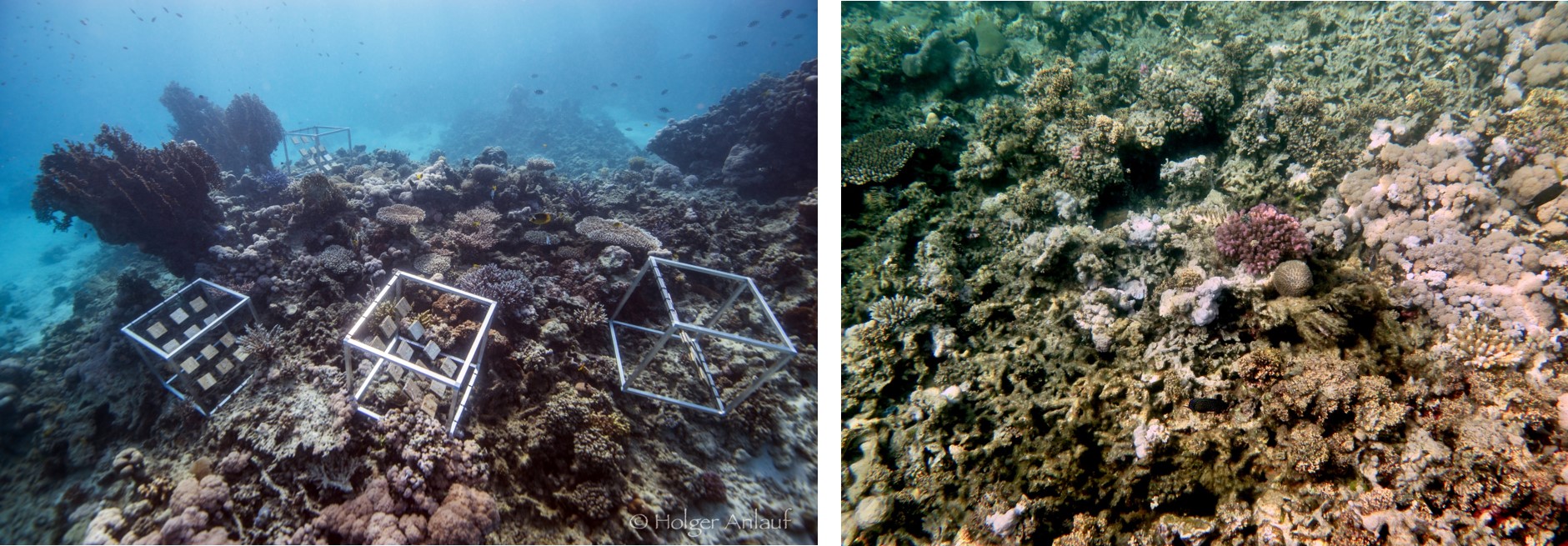


Figure S1: Photos of investigated reef areas showing distinct coral-dominated (left) and algae-dominated reef areas (right). Right picture was taken by Holger Anlauf and was initially published in Roth and others (2018)^1^, right photo was taken by Florian Roth.

**SM 2 – Relative benthic cover in coral- and algae-dominated reef areas**

Table S1: Relative benthic cover in coral- and algae-dominated reef areas. Presented are mean proportional cover in % ± standard error of major benthic categories assessed by photo quadrats (n = 10 in coral-dominated areas, n = 12 in algae-dominated areas).

| Benthic category | Relative cover [%] in coral-dominated area | Relative cover [%] in algae-dominated area |
| --- | --- | --- |
| Hard Coral | 43.55 ± 5.67 | 13.96 ± 2.33 |
| Soft Coral (*Xenia* sp.) | 8.85 ± 3.78 | 8.13 ± 1.68 |
| Turf Algae | 26.15 ± 5.02 | 47.50 ± 7.15 |
| Biogenic Rock | 16.11 ± 3.05 | 1.25 ± 0.71 |
| Coral Rubble | 3.30 ± 3.11 | 12.08 ± 5.75 |
| Reef Sands | 0.52 ± 0.27 | 11.46 ± 3.66 |
| *Tridacna* | 1.34 ± 0.82 | 0.00 ± 0.00 |
| Macro Algae | 0.17 ± 0.00 | 0.63 ± 0.32 |
| Sponges | 0.00 ± 0.00 | 0.00 ± 0.00 |

**SM 3 – Dinitrogen (N_2_) fixation rates and denitrification potentials**

Table S2: Dinitrogen (N_2_) fixation rates and denitrification potentials of investigated benthic categories. Rates are presented in mean ± standard errors and in pmol N_2_ cm^-2^ h^-1^. Data for hard corals consists of mean values of P. verrucosa, A. hemprichii and S. pistillata.

| Benthic Category | Replicates | N_2_ fixation | Denitrification |
| --- | --- | --- | --- |
| Turf Algae | 5 | 570.30 ± 59.18 | 6.01 ± 1.10 |
| Coral Rubble | 4 | 482.07 ± 88.40 | 12.47 ± 1.29 |
| Soft Coral (*Xenia* sp.) | 5 | 14.62 ± 4.16 | 36.79 ± 9.02 |
| Biogenic Rock | 5 | 97.77 ± 15.99 | 13.64 ± 1.52 |
| Reef Sands | 5 | 45.69 ± 6.07 | 11.25 ± 3.01 |
| Hard Corals | 13 | 5.11 ± 1.89 | 6.47 ± 2.27 |
| *Pocillopora verrucosa* | 5 | 2.52 ± 0.42 | 3.71 ±0.77 |
| *Acropora hemprichii* | 4 | 6.57 ± 3.23 | 15.80 ± 4.56 |
| *Stylophora pistillata* | 4 | 6.88 ± 4.86 | 0.59 ± 0.25 |

**SM 4 – Description of incubated benthic categories with respective 2D to 3D conversion factor**

Table S3: Description of incubated benthic categories, and respective 2D to 3D conversion factor according to Cardini and others (2016)^17^.

| Benthic category | 2D to 3D conversion factor | Description/incubated organism or substrate |
| --- | --- | --- |
| Hard Corals | 7.6 | *Pocillopora, Acropora, Stylophora* |
| Soft Coral | 6.5 | Xeniidae |
| Biogenic Rock | 2.5 | Biogenic reef framework with visible carbonate structure (according to Bahartan and others (2010)^18^ |
| Coral Rubble | 5.2 | Equal to coral rock, uncovered dead coral skeleton according to Rasser and Riegl (2002)^19^ |
| Turf Algae | 3.9 | Dense mats composed of a heterogeneous assemblage of filamentous algae |
| Reef Sands | 1.0 | Surface layer with its associated microphytobenthos |

**SM 5 – Nitrate concentration quantifications**

Nitrate (NO_3_^-^) was added during the N cycling incubations serving as a substrate for denitrification, as the natural formulation pathway (nitrification) of NO_3_^-^ is inhibited by acetylene ^5–7^. NO_3_^-^ was added in form of a previously prepared sodium nitrate (NaNO_3_) stock solution, prepared with MilliQ water and NaNO_3_ ≥99.0% (Sigma Aldrich). Water samples of incubation water were taken at the beginning of each incubation. Water samples were filtered immediately (Isopore^TM^ GTTP membrane filters, 0.2 µm) and the filtrate was stored frozen at -50°C in the lab after collection. NO_3_^-^ concentrations as well as further parameters (nitrite (NO_2_^-^) and phosphate (PO_4_^3-^)) were determined using a continuous flow analyser (AA3, HR, SEAL, following colourimetric standard methods ^8^). Limits of quantification for NO_3_^-^ NO_2_^-^ and PO_4_^3-^ were 0.084, 0.011 and 0.043 µmol L^-1^, respectively. Mean initial concentrations (n = 4) as well as seawater control concentrations of NO_3_^-^ NO_2_^-^ and PO_4_^3-^ are displayed in Tab. S2.

Table S4: Mean ± SE values (n = 4) of nitrate (NO_3_^-^), nitrite (NO_2_^-^) and phosphate (PO_4_^3-^) at the start of nitrogen cycling incubations (i.e., amended with 5 µM nitrate) and seawater controls

| Parameter | µmol L^-1^ incubation chamber | µmol L^-1^ seawater control |
| --- | --- | --- |
| NO_3_^-^ | 5.743 ± 0.17 | 1.056 ± 0.064 |
| NO_2_^-^ | 0.071 ± 0.027 | 0.060 ± 0.008 |
| PO_4_^3-^ | 0.116 ± 0.025 | 0.126 ± 0.009 |

**SM 6 – Calculation of N_2_ fixation and denitrification rates according to El-Khaled et al. (2020)**^9^

The measured headspace concentrations of ethylene (C_2_H_4_) and nitrous oxide (N_2_O), respectively, which were quantified via gas chromatography (GC), were used to calculate the total dissolved amount of C_2_H_4_ and N_2_O in the incubation chamber. The total amount of C_2_H_4_ and N_2_O in the incubation chamber can be calculated via the addition of headspace and incubation water. These values can be used to approximate N_2_ fixation and denitrification rates/potentials^10,11^. The calculation process can be described as follows:

The calculation of the absolute amount of C_2_H_4_ in the incubation chamber headspace (C_2_H_4_HS_; [nM]) can be described according to the following equation:

(1) $C_{2}H_{4\_HS}=R^{-1}*C_{2}H_{4\_ms}*{Vol}_{HS}*{10}^{3}$

With:

*R*  ideal gas constant; defined as 24,465 cm^3^ mol^-1^ at 25 ˚C in our case as both gases act identical to ideal gases;

C_2_H_4_ms_ measured headspace concentration [ppm]; possible equilibrium transformations (Boyle-Mariotte law, according to Job and Rüffler (2016)^12^) have to be considered depending on the volume of the syringes used for sampling and transferring the gaseous sample from collection tube to the GC and the volume of the collection tube itself (here: 2.5 mL);

*Vol_HS_* headspace volume [cm^3^].

10^3^ result of cancelations from unit-transformations for ppm to mol conversion

Accordingly, the total C_2_H_4_ amount in the incubation water (C_2_H_4_IW_; [nM]) was calculated according to:

(2) $C_{2}H_{4\_IW}=R^{-1}*C_{2}H_{4\_ms}*\beta*{Vol}_{IW}*{10}^{3}$

With:

*β* Bunsen-solubility coefficient for C_2_H_4_ according to Breitbarth et al. (2004)^13^;

*Vol_IW_* incubation water volume [mL].

The total C_2_H_4_ amount of the incubation chamber (C_2_H_4_tot_; [nM]) can be calculated with a simple addition as:

(3) $C_{2}H_{4\_tot}=C_{2}H_{4\_HS}+C_{2}H_{4\_IW}$

To ensure comparability between different specimens, rates should be normalized to either surface area (SA; e.g., by creating 3D models of the specimens with computer software) or dry weight (DW)^2,3,10^. To calculate for metabolic background activities, rates of control incubations/seawater blanks have to be considered at this point too. In addition, rates should also be normalized to incubation time (e.g., rates per hour, rates per day, etc.). These two reference parameters (SA or DW, time) can be included according to the following equation, resulting in comparable rates between replicates (C_2_H_4_rate_; [nM time^-1^ SA^-1^ or nM time^-1^ g^-1^ DW]):

(4) $C_{2}H_{4\_rate}= \frac{\left( C_{2}C_{4\_tot\_smpl\_tx}- C_{2}H_{4\_tot\_smpl\_t0} \right)-(C_{2}H_{4\_tot\_ctrl\_tX}-C_{2}H_{4\_tot\_ctrl\_t0})}{\left( t_{x}-t_{0} \right)*RP}$

With:

C_2_H_4_tot_smpl_tX_ C_2_H_4_tot_ at t_X;_

C_2_H_4_tot_smpl_t0_ C_2_H_4_tot_ at t_0;_

C_2_H_4_tot_ctrl_tX_  C_2_H_4_tot_ of control incubation/seawater blank at t_X;_

C_2_H_4_tot_ctrl_t0_ C_2_H_4_tot_ of control incubation/seawater blank at t_0;_

*RP* reference parameter (i.e., SA or DW).

A transformation from C_2_H_4_ evolution to N_2_ fixation rates could be considered by using accurate conversion factors according to Mulholland et al. (2004)^14^ or Charpy-Roubaud et al. (2001)^15^.

The calculations for the total N_2_O concentration can be performed similarly to the approach presented before with the annotation of considering a different solubility factor *β* ^16^ and neglecting a conversion factor for N_2_O for the quantification of total denitrification*.*

**SM 7 – Quantification of oxygen concentrations during the incubation period**

Oxygen fluxes were quantified for parallel studies with identical benthic categories (unpubl. data) that were sampled <3 h before oxygen flux incubations. Incubation chambers (1L volume) were filled with ambient seawater that was collected from the same day. Specimens (n = 5 for reef sands, *Xenia* sp., turf algae, coral rubble, biogenic rock, and *P. verrucosa*; n = 4 for *Acropora damicornis*, and *Stylophora pistillata*; n = 6 control chambers to correct for background metabolism) were placed inside the incubation chambers. All chambers were sealed gastight and without any air enclosure. Incubation chambers were placed in a tempered water bath and constantly stirred (500 rpm) to ensure stable measurement conditions (27°C). A 2 h light (~200 µM quanta m^-2^ s^-1^ photon flux) was performed prior a 2 h dark incubation with fresh ambient seawater. Oxygen levels were measured immediately before starting the respective incubation and after 2 h using a WTW Multi 3430 which was equipped with a WTW DFO 925 oxygen sensor. Results were normalised to incubation time and surface area (surface areas of hard and soft corals, turf algae, biogenic rock, coral rubble were calculated using cloud-based 3-dimensional models of samples ^2,3^, reef sands were mathematically calculated). Oxygen rates for each benthic category were then used to extrapolate the oxygen concentrations at respective time points during nitrogen incubations, considering the respective surface area of the specimens as well as the given headspace (200mL headspace – 20mL that was exchanged for acetylene addition). Results for extrapolated oxygen concentrations over time in the incubation chambers for N cycling quantifications are presented in Tab. S1. Our results show that thresholds for hypoxia (i.e., condition of low dissolved oxygen that becomes detrimental to aerobic organisms) and hyperoxia (i.e., condition where oxygen exceeds 100% air saturation) were not exceeded (see Hughes et al., 2020). We, thus, believe that both N cycling pathways were assessed in representative scenarios.

Table S5: Extrapolated range of hourly minimum and maximum oxygen (O_2_) concentrations in the incubation water during nitrogen (N) cycling incubation

| Benthic category | Range of mean O_2_ concentrations in incubation water [mg L^-1^] during N cycling incubation |
| --- | --- |
| Turf Algae | 5.00 – 6.49* |
| Coral Rubble | 5.37 – 6.43* |
| Soft Coral (*Xenia* sp.) | 5.52 – 6.43* |
| Biogenic Rock | 5.51 – 6.44 |
| Reef Sands | 5.85 – 6.50* |
| *Pocillopora verrucosa* | 4.33 – 6.50* |
| *Acropora hemprichii* | 5.52 – 6.50* |
| *Stylophora pistillata* | 4.82 – 6.49* |

* = indicates that the starting concentration was not exceeded during incubation

**SM 8 – Formulas of extrapolated fixed/removed N per benthos 3D area**

(A) $N_{F}= \sum{measured rate}_{i}*{2D to 3D cf}_{i}*{benthic cover}_{i}$

(B) $N_{R}= \sum{measured rate}_{i}*{2D to 3D cf}_{i}*{benthic cover}_{i}$

With: N_F_ fixed nitrogen via dinitrogen fixation

N_R_ removed nitrogen via denitrification

Measured rate_i_ quantified N_2_ fixation/denitrification rate of respective benthic category according to table S2

2D to 3D cf_i_ 2D to 3D conversion factor of respective benthic category according to table S3

Benthic cover_i_ benthic cover of respective benthic category according to table S1

**References**

1. Roth, F. *et al.* Coral reef degradation affects the potential for reef recovery after disturbance. *Mar. Environ. Res.* **142**, 48–58 (2018).

2. Gutierrez-Heredia, L., Benzoni, F., Murphy, E. & Reynaud, E. G. End to End Digitisation and Analysis of Three-Dimensional Coral Models, from Communities to Corallites. *PLoS One* **11**, e0149641 (2016).

3. Lavy, A. *et al.* A quick, easy and non-intrusive method for underwater volume and surface area evaluation of benthic organisms by 3D computer modelling. *Methods Ecol. Evol.* **6**, 521–531 (2015).

4. Hughes, D. J. *et al.* Coral reef survival under accelerating ocean deoxygenation. *Nat. Clim. Chang.* **10**, 296–307 (2020).

5. Haines, J. R., Atlas, R. M., Griffiths, R. P., Morita, R. Y. & Sea, B. Denitrification and Nitrogen Fixation in Alaskan Continental Shelf Sediments. **41**, 412–421 (1981).

6. Joye, S. B. & Paerl, H. W. Contemporaneous nitrogen fixation and denitrification in intertidal microbial mats: rapid response to runoff events. *Mar. Ecol. Prog. Ser.* **94**, 267–274 (1993).

7. Miyajima, T., Suzumura, M., Umezawa, Y. & Koike, I. Microbiological nitrogen transformation in carbonate sediments of a coral-reef lagoon and associated seagrass beds. *Mar. Ecol. Prog. Ser.* **217**, 273–286 (2001).

8. Grasshoff, K., Kremling, K. & Ehrhardt, M. *Methods of seawater analysis*. (Wiley-VCH, 1999).

9. El-Khaled, Y. C. *et al.* Simultaneous Measurements of Dinitrogen Fixation and Denitrification Associated With Coral Reef Substrates : Advantages and Limitations of a Combined Acetylene Assay. *Front. Mar. Sci.* **7**, 411 (2020).

10. Haines, J. R., Atlas, R. M., Griffiths, R. P. & Morita, R. Y. Denitrification and Nitrogen Fixation in Alaskan Continental Shelf Sediments. *Appl. Environ. Microbiol.* **41**, 412–421 (1981).

11. Wilson, S. T., Böttjer, D., Church, M. J. & Karl, D. M. Comparative assessment of nitrogen fixation methodologies, conducted in the oligotrophic north pacific ocean. *Appl. Environ. Microbiol.* **78**, 6516–6523 (2012).

12. Job, G. & Rüffler, R. Molecular-Kinetic View of Dilute Gases. in *Physical Chemistry from a Different Angle* 271–294 (Springer, Cham, 2016). doi:10.1007/978-3-319-15666-8

13. Breitbarth, E., Mills, M. M., Friedrichs, G. & Laroche, J. The Bunsen gas solubility coefficient of ethylene as a function of temperature and salinity and its importance for nitrogen fixation assays. *Limnol. Oceanogr. Methods* **2**, 282–288 (2004).

14. Mulholland, M. R., Bronk, D. A. & Capone, D. G. Dinitrogen fixation and release of ammonium and dissolved organic nitrogen by Trichodesmium IMS101. *Aquat. Microb. Ecol.* **37**, 85–94 (2004).

15. Charpy-Roubaud, C., Charpy, L. & Larkum, A. Atmospheric dinitrogen fixation by benthic communities of Tikehau lagoon (Tuamotu Archipelago, French Polynesia) and its contribution to benthic primary production. *Mar. Biol.* **139**, 991–997 (2001).

16. Weiss, R. F. & Price, B. A. Nitrous Oxide Solubility in Water and Seawater. *Mar. Chem.* **8**, 347–359 (1980).

17. Cardini, U. *et al.* Budget of Primary Production and Dinitrogen Fixation in a Highly Seasonal Red Sea Coral Reef. *Ecosystems* **19**, 771–785 (2016).

18. Bahartan, K. *et al.* Macroalgae in the coral reefs of Eilat (Gulf of Aqaba, Red Sea) as a possible indicator of reef degradation. *Mar. Pollut. Bull.* **60**, 759–764 (2010).

19. Rasser, M. W. & Riegl, B. Holocene coral reef rubble and its binding agents. *Coral Reefs* **21**, 57–72 (2002).
